# Molecular Basis for Stage-Specific Host Preference in the Aphid Parasitoid *Binodoxys communis*

**DOI:** 10.3390/insects16111127

**Published:** 2025-11-04

**Authors:** Tingfang Zhong, Cen Bai, Jinming Li, Li Wang, Kaixin Zhang, Dongyang Li, Jichao Ji, Xiangzhen Zhu, Xueke Gao, Weihua Ma

**Affiliations:** 1 Zhengzhou Research Base, State Key Laboratory of Cotton Bio-Breeding and Integrated Utilization, Institute of Cotton Research, Chinese Academy of Agricultural Sciences, Anyang 455000, China; zhong_tingfang@163.com (T.Z.); 19527513042@163.com (C.B.); lijinmingzzu@163.com (J.L.); wangli08zb@126.com (L.W.); zhangkaixin@caas.cn (K.Z.); hzaulidongyang@163.com (D.L.); hnnydxjc@163.com (J.J.); 2 College of Plant Science and Technology, Huazhong Agricultural University, Wuhan 430070, China; 3 State Key Laboratory of Cotton Bio-Breeding and Integrated Utilization, School of Agricultural Sciences, Zhengzhou University, Zhengzhou 450001, China

**Keywords:** *Aphis gossypii*, *Binodoxys communis*, reproductive manipulation, stage-specific parasitism, transcriptomics

## Abstract

**Simple Summary:**

The cotton aphid is a major global pest, damaging crops through its explosive reproduction. A tiny wasp, *Binodoxys communis*, shows promise as a natural control method but scientists do not fully understand how it manipulates the aphid’s reproduction across different host stages. Our research reveals that parasitism by the wasp impairs aphid development and severely suppresses reproduction, with the most pronounced effects on younger aphids. We discovered that silencing a single master regulator gene is sufficient to control young aphids, whereas suppressing reproduction in adults requires the coordinated downregulation of multiple genes. This explains why the wasp favors nymphs—an efficient “low-cost, high-reward” strategy honed by evolution. These insights deepen our grasp of nature’s pest battles and pinpoint genes for better eco-friendly farming tools, helping farmers protect crops without the use of harsh chemicals.

**Abstract:**

The cotton aphid *Aphis gossypii* is a globally significant agricultural pest that threatens crop production through its prolific reproduction. While the parasitoid wasp *Binodoxys communis* offers promising potential for biological control, the molecular mechanisms underlying its reproductive manipulation of aphid hosts remain poorly understood. Here, we investigated the stage-specific parasitism strategies of *B. communis* on *A. gossypii* using integrated biological observations and transcriptomic analysis. Parasitism significantly prolonged aphid development and suppressed reproduction across all host stages, with severity inversely correlated with host age at parasitism. Transcriptomic analysis of ovaries of parasitized aphids revealed 1168 differentially expressed genes, with temporal progression from minimal changes in nymphs (7 DEGs at day 1) to extensive disruption in adults (549 DEGs at day 3). Notably, juvenile hormone acid methyltransferase (*JHAMT*), the rate-limiting enzyme in juvenile hormone biosynthesis, emerged as a master regulator that is differentially targeted across host stages. In 3rd instar nymphs, single-gene suppression of *JHAMT* (−3.23-fold change) achieved effective reproductive control, whereas adult parasitism required progressive manipulation of multiple genes including *JHAMT*, *FOHSDR*, *ALDH*, and *JHEH*. The vitellogenin-vitellogenin receptor system only showed coordinated downregulation in adults, whereas nymphs exhibited preemptive receptor suppression before vitellogenesis onset. These findings demonstrate that *B. communis* has evolved to exploit a developmental window where host manipulation is most efficient—3rd instar nymphs, which possess sufficient resources for parasitoid development and lack the complex compensatory mechanisms found in adults. This “low-cost, high-reward” strategy based on precision targeting of master regulators in nymphs compared to multi-gene assault in adults, revealing the evolutionary optimization of parasitoid manipulation strategies. Our results provide molecular insights into parasitoid-host coevolution and identified key regulatory targets for developing innovative biological control strategies against this important agricultural pest.

## 1. Introduction

*Aphis gossypii* Glover (Hemiptera: Aphididae) is a highly adaptable and prolific pest with a worldwide distribution that causes serious damage to cotton production [1,2,3]. In recent years, large-scale outbreaks of this aphid have resulted in substantial economic losses [4]. The challenges of effective aphid management are further compounded by climate change, crop diversification, and agricultural intensification, necessitating the development of innovative control strategies. *Binodoxys communis* Gahan (Hymenoptera: Braconidae), a koinobiont endoparasitoid, is a promising biological control agent for various aphid species, including cotton, corn, and soybean aphids [5,6,7]. Parasitoid wasps orchestrate host manipulation through four primary mechanisms mediated by venom proteins and peptides: suppression of host immunity [8,9,10,11,12,13], growth and development [14,15,16,17], nutrient metabolism [18,19,20], and reproduction [21,22,23,24]. Among these, reproductive manipulation represents a particularly effective strategy, as it directly eliminates host population growth while preserving host resources for parasitoid development [25].

Parasitoids exhibit selective host preferences influenced by developmental stage traits, reflecting an evolutionary optimization strategy in which they exploit developmental windows for efficient manipulation through minimal molecular intervention [26,27,28,29,30]. Although some aphidiid parasitoids can oviposit in all host stages, including adults, they typically exhibit a preference for 2nd–3rd or 2nd–4th instar nymphs [31,32]. Despite extensive research on parasitoid-induced alterations in host immunity, development, and metabolism [6,33,34,35,36], the molecular mechanisms governing stage-specific strategies, particularly how *B. communis* regulates the reproductive capacity of *A. gossypii*, remain largely unexplored. The ovary, as the primary reproductive organ, serves as an ideal system for investigating parasitoid-mediated reproductive manipulation at the molecular level. Understanding the transcriptomic changes in ovaries of parasitized aphids could reveal key regulatory targets and clarify the molecular basis for the efficiency of stage-specific manipulation strategies.

In this study, we investigated the impact of *B. communis* parasitism on the development and reproduction of *A. gossypii* across different host stages. Through comprehensive transcriptomic analysis of ovarian tissues from parasitized and non-parasitized aphids, we aimed to: (1) characterize the molecular mechanisms underlying reproductive suppression; (2) identify stage-specific gene expression patterns that explain parasitoid host preferences; and (3) elucidate the molecular basis of parasitoid preference for specific host stages. These findings will enhance the understanding of parasitoid-host coevolution and provide theoretical foundation for optimizing biological control strategies in agricultural systems.

## 2. Materials and Methods

### 2.1. Insects and Plants

The cotton aphid, *Aphis gossypii* Glover (Hemiptera: Aphididae), was reared parthenogenetically at the Cotton Research Institute of the Chinese Academy of Agricultural Sciences (36°5′34.8″ N, 114°31′47.19″ E) under controlled conditions of 26.0 ± 1 °C, 65 ± 5% relative humidity (RH), and a 16:8 h (light: dark) photoperiod. The parasitoid wasp *Binodoxys communis* Gahan (Hymenoptera: Braconidae) was maintained under identical conditions. Cotton plants (*Gossypium hirsutum* cv. Zhongmian49) from the Mid-term Database of Cotton Germplasm Resources at the same institute were used as host plants and were cultivated at 26.0 ± 1 °C, 70 ± 5% RH, and a 14:10 h (light: dark) photoperiod.

### 2.2. The Growth and Development of A. gossypii in Response to the Parasitism by B. communis

To assess the impact of parasitism by *B. communis* on the growth and development of *A. gossypii*, wingless aphids at various developmental stages (1st, 2nd, 3rd, and 4th instars) were placed on glass dishes lined with cotton leaves. Mated female *B. communis* were introduced for parasitization, and a cotton ball soaked in honey water was provided as a food source. Non-parasitized aphids of the same age served as controls. Parasitism was confirmed by observing the stinging behavior. Subsequently, both parasitized and control aphids were transferred to an agar-based culture system with fresh cotton leaves for further observation. Only aphids that successfully developed into mummies were recorded as parasitized and used for subsequent data analysis. Three replicates were established, each containing 20 aphids. The developmental stages of both groups were recorded daily.

### 2.3. The Reproduction of A. gossypii in Response to the Parasitism by B. communis

The effect of parasitism on the reproductive capacity of *A. gossypii* was evaluated using a similar experimental setup as described in Section 2.2. Parasitized and non-parasitized wingless aphids were monitored daily for offspring production. The experiment was conducted in 3 replicates, each with 20 aphids.

### 2.4. Ovary Dissection and RNA Extraction

Wingless 3rd instar nymphs and adults *A. gossypii*, both parasitized and non-parasitized by *B. communis*, were utilized for ovary dissection. Parasitism was confirmed by observing the stinging behavior, and successfully parasitized aphids were promptly removed. Both the parasitized aphids and the non-parasitized aphids were maintained on a cotton leaf under the same incubator conditions. Ovary dissection was performed under a microscope after 1 and 3 days of parasitism for 3rd instar nymphs and adults. Only aphid samples in which parasitoid larvae were confirmed present during dissection were processed for RNA extraction and sequencing. Ovaries from non-parasitized aphids of the same age was dissected as controls. The collected samples were frozen in liquid nitrogen and stored at −80 °C until RNA extraction for transcriptome sequencing. Three replicates were prepared, each containing 60 ovaries. Total RNA was extracted from the ovaries of both parasitized and non-parasitized *A. gossypii* using the TRIzol method. RNA concentration and purity were assessed using a Nanodrop 2000 spectrophotometer (Thermo Scientific, Waltham, MA, USA), and RNA integrity was verified by 1.5% agarose gel electrophoresis. Only samples with A_260_/A_280_ ratios between 1.8–2.0 and clear 28S/18S rRNA bands were used for sequencing.

### 2.5. Library Preparation and Transcriptome Sequencing

Poly(A)+ mRNA was isolated from total RNA using oligo(dT) magnetic beads. Purified mRNA was fragmented to ~300 bp using fragmentation buffer. First-strand cDNA was synthesized using random hexamer primers and reverse transcriptase, followed by second-strand synthesis to generate double-stranded cDNA. Libraries were prepared by end-repair, A-tailing, and adapter ligation. Sequencing was performed on an Illumina NovaSeq 6000 platform (Majorbio, Shanghai, China), generating 150 bp paired-end reads.

### 2.6. Data Processing and Analysis

Raw reads were processed using fastp v0.19.5 (https://github.com/OpenGene/fastp, accessed on 13 December 2024) to remove adapters, low-quality bases (Q < 30), ambiguous nucleotides (>10% N), and short reads (<50 bp). Clean reads were mapped to the *A. gossypii* reference genome using HISAT2 v2.1.0 (http://ccb.jhu.edu/software/hisat2/index.shtml, accessed on 18 December 2021) with default parameters. Transcript assembly and abundance quantification were performed using StringTie v2.1.2 (https://ccb.jhu.edu/software/stringtie/, accessed on 21 December 2021) and RSEM v1.3.3, respectively. Gene expression was normalized as FPKM (Fragments Per Kilobase per Million mapped reads). Genes with FPKM > 1 were retained for analysis. Differential expression analysis was conducted using DESeq2 with thresholds of |log_2_(fold change)| ≥ 1 and adjusted *p*-value < 0.05. Functional enrichment analyses were performed using GOatools v0.6.5 for Gene Ontology and custom R scripts for KEGG pathways. Enrichment significance was determined using Fisher’s exact test with Benjamini–Hochberg correction (P-adjust < 0.05).

### 2.7. RT-qPCR to Assess Gene Expression Change

Seven reproduction-related differentially expressed genes (DEGs) were selected for validation. Gene-specific primers were designed using Primer 6 and synthesized by Sangon Biotech (Shanghai, China) (Table 1). RT-qPCR was performed using SYBR Green chemistry with *β-actin* serving as the reference gene for normalization. Relative expression was calculated using the 2^−ΔΔCT^ method [37]. Three biological replicates with three technical replicates each were analyzed.

### 2.8. Statistical Analysis

Data were analyzed using SPSS version 20 (IBM Corp., Armonk, NY, USA). Differences between parasitized and control groups were assessed using the appropriate statistical tests (e.g., Student’s *t*-test or ANOVA), with the significance threshold set at *p* < 0.05. Confidence intervals (CIs) were calculated at 95%. Figures were generated using GraphPad Prism version 9 (GraphPad Software, San Diego, CA, USA) and the Majorbio cloud platform (https://www.majorbio.com, accessed on 20 May 2025).

## 3. Results

### 3.1. The Growth and Development of A. gossypii in Response to the Parasitism by B. communis

**Figure 1:**
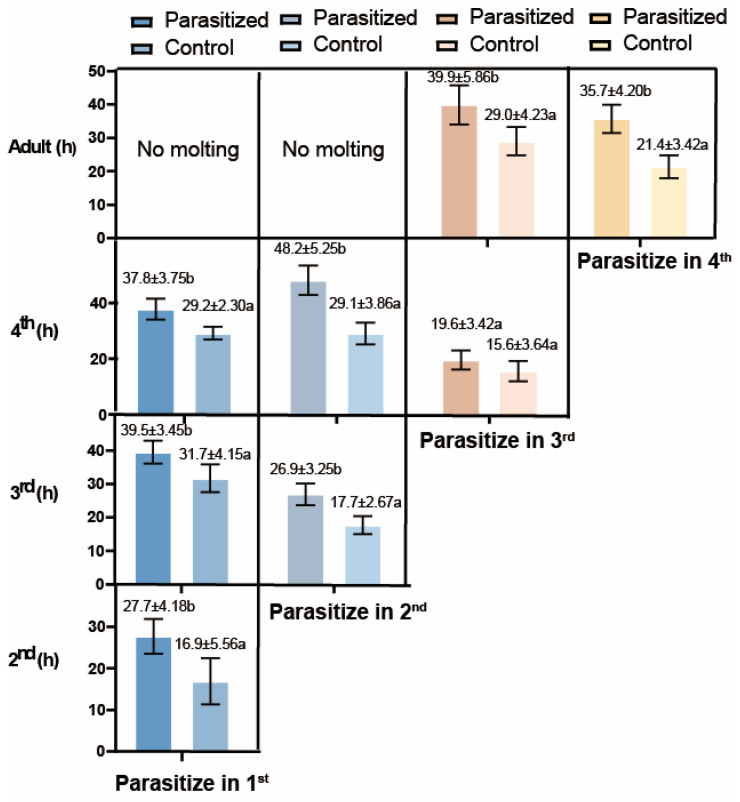
Developmental durations at different host stages [mean ± standard error (number of individuals that completed the stage)]. Letters indicate the significance of the same parasitic days at the same age (T tests with CIs = 95%).

As shown in Figure 1, parasitism by *B. communis* significantly delayed the growth and development of *A. gossypii* compared to non-parasitized controls. The duration of the 4th instar stage was extended by 19.1 h when aphids were parasitized as 2nd instars, and by 4 h when parasitized as 3rd instars. Parasitism consistently prolonged developmental times from 1st to 2nd, 3rd and 4th instars (from 16.9, 31.7, and 29.2 h to 27.7, 39.5, and 37.8 h, respectively), with no molting observed beyond the 4th instar. Regardless of the initial instar (1st, 2nd, 3rd, or 4th), parasitized *A. gossypii* nymphs consistently exhibited prolonged developmental durations across all instars (2nd to adult), with all stages demonstrating similar extension trends. These results indicated that parasitism by *B. communis* significantly impaired the growth and development of *A. gossypii*.

### 3.2. Reproduction of A. gossypii in Response to the Parasitism by B. communis

**Figure 2:**
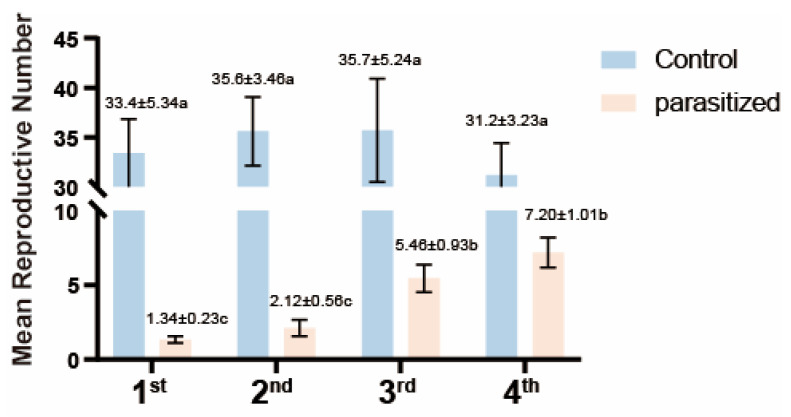
The offspring production of *Aphis gossypii* in Response to Parasitism by *Binodoxys communis* [mean ± standard error means reproductive number (±SD) per generation]. Different letters indicate statistically significant differences between treatments within the different generations (Tukey’s HSD test with CIs = 95%).

The fecundity of *A. gossypii* was significantly reduced by *B. communis* compared to the non-parasitized individuals, with reduction severity dependent on host developmental stage. The maximum reduction occurred in 1st instar, while minimal reduction occurred in 4th instar. After parasitization, the average number of offspring numbers of 1st, 2nd, 3rd, and 4th aphids were 1.34, 2.12, 5.46, and 7.20, respectively, while the average number of offspring of non-parasitized aphids were 33.4, 35.6, 35.7, and 31.2 (Figure 2).

### 3.3. Transcriptome Sequencing and Differential Expression Analysis

**Figure 3:**
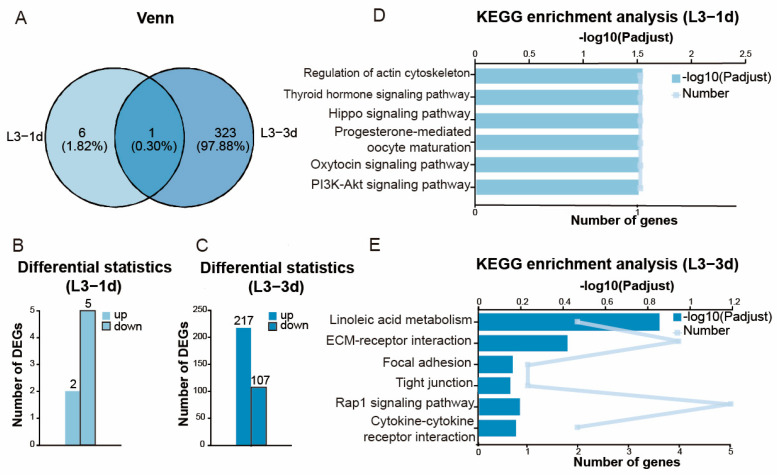
Differential gene expression and KEGG enrichment analysis of ovaries of 3rd instar *Aphis gossypii* nymphs following parasitism by *Binodoxys communis*. (**A**) Venn diagram of differentially expressed genes (DEGs) in 3rd instar *A. gossypii* nymphs parasitized by *B. communis* on day 1 (L3-1d) vs. day 3 (L3-3d). Data annotations indicate gene counts and proportions. (**B**) Number of DEGs after the nymphs were parasitized for 1 day. (**C**) Number of DEGs after the nymphs were parasitized for 3 days. (**D**) KEGG enrichment analysis after the nymphs were parasitized for 1 day. (**E**) KEGG enrichment analysis after the nymphs were parasitized for 3 days.

**Figure 4:**
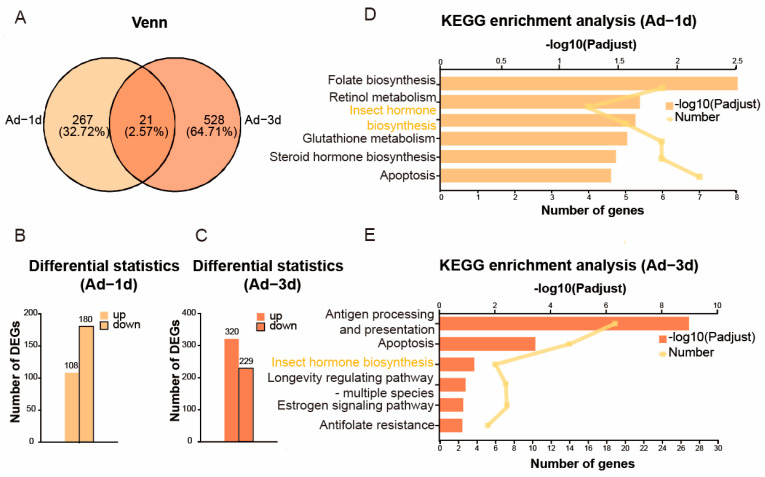
Differential gene expression and KEGG enrichment analysis of ovaries of *Aphis gossypii* adults following parasitism by *Binodoxys communis*. (**A**) Venn diagram of differentially expressed genes (DEGs) in *A. gossypii* adults parasitized by *B. communis* on day 1 (Ad-1d) vs. day 3 (Ad-3d). Data annotations indicate gene counts and proportions. (**B**) Number of DEGs after the adults were parasitized for 1 day. (**C**) Number of DEGs after the adults were parasitized for 3 days. (**D**) KEGG enrichment analysis after the adults were parasitized for 1 day. (**E**) KEGG enrichment analysis after the adults were parasitized for 3 days.

A total of 155.53 Gb of clean data were generated after quality filtering, (≥6.03 Gb/sample), with a GC content ranging from 32.57% to 36.29%. Data reliability was confirmed by Q20 (>97%) and Q30 (>91.6%) scores. A total of 1168 differentially expressed genes (DEGs) were identified across all comparisons between parasitized and non-parasitized samples. Specifically, after the 3rd instar nymphs were parasitized for 1 day (Figure 3A,B), 7 DEGs were found (2 upregulated, 5 downregulated). After the 3rd instar nymphs were parasitized for 3 days (Figure 3A,C), 324 DEGs were identified (217 upregulated, 107 downregulated). After the adults were parasitized for 1 day (Figure 4A,B), 288 DEGs were detected (108 upregulated, 180 downregulated). After the adults were parasitized for 3 days (Figure 4A,C), 549 DEGs were observed (320 upregulated, 229 downregulated).

### 3.4. KEGG Pathway Analysis

KEGG pathway analysis revealed key pathways implicated in the reproductive regulation affected by parasitism. Most notably, the insect hormone biosynthesis pathway was significantly enriched after the adults were parasitized for 1 and 3 days, representing a primary target through which parasitoids manipulate host reproduction (Figure 4D,E), but this pathway was not enriched after the 3rd instar nymphs were parasitized for 1 day (Figure 3D) and showed no significant enrichment (*p* = 0.80) after the 3rd instar nymphs were parasitized for 3 days (Figure 3E). The pathway controls the synthesis of juvenile hormone and ecdysone, master regulators of insect reproduction and development. Additionally, the activation of apoptosis pathways after parasitizing for 1 and 3 days in adults provided direct molecular evidence for programmed cell death in ovarian tissues, which is likely responsible for the reproductive failure (Figure 4D,E). Furthermore, after the adults were parasitized for 3 days, activation of antigen-processing and presentation pathways (Figure 4E) suggested a reallocation of host resources from reproduction to immune responses.

### 3.5. JHAMT-Centered Gene Regulation Underlies Parasitoid Host Preference

**Figure 5:**
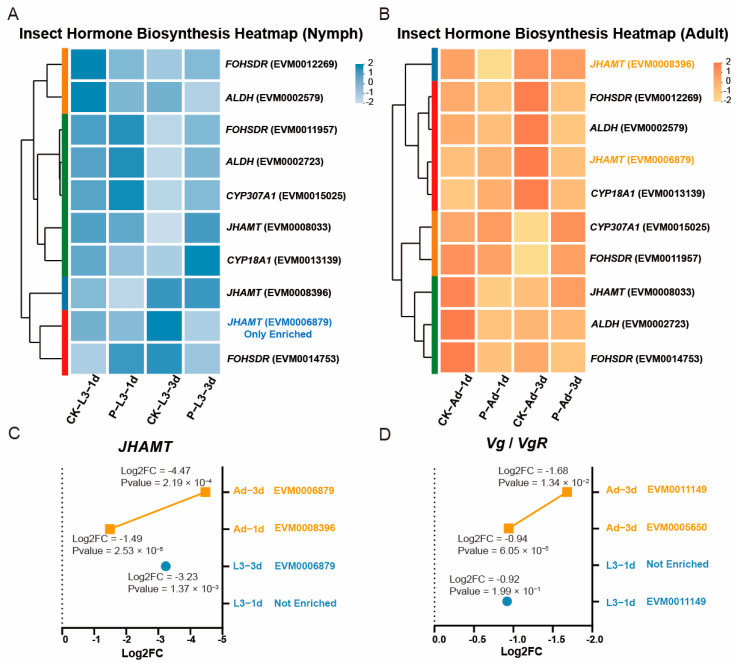
Expression patterns of key reproductive regulatory genes in *Aphis gossypii* ovaries following *Binodoxys communis* parasitism. (**A**) Heatmap showing expression profiles of genes related in the insect hormone biosynthesis pathway in nymphs. (**B**) Heatmap showing expression profiles of genes enriched in the insect hormone biosynthesis pathway in adults. (**C**). Log2FC of *JHAMT* expression at different parasitism stages. (**D**) Log2FC of vitellogenin (*Vg*) and vitellogenin receptor (*VgR*) expression. Annotation and Explanation: P and CK represent parasitized and control samples; L3-1d and L3-3d represent 3rd instar nymphs parasitized for 1 and 3 days; Ad-1d and Ad-3d represent adults parasitized for 1 and 3 days.

Among the 10 genes identified in the insect hormone biosynthesis pathway (Figure 5A,B), *JHAMT* (juvenile hormone acid methyltransferase) emerged as the most consistently affected target across all developmental stages and time points, although its manipulation differed dramatically between host stages. In 3rd instar nymphs, *JHAMT* was the sole hormone synthesis gene that was significantly downregulated (Log2FC = −3.23, *p* = 1.37 × 10^−3^) after parasitization for 3 days, achieving reproductive disruption through minimal molecular intervention. This contrasts sharply with adult parasitism, where *JHAMT* downregulation intensified progressively from −1.49 (*p* = 2.53 × 10^−5^) on day 1 to −4.47 (*p* = 2.19 × 10^−4^) on day 3 (Figure 5C), accompanied by the concurrent suppression of multiple other pathway components including *FOHSDR*, *ALDH*, and *JHEH* (Figure 5B). In contrast to the multi-gene suppression required for adults, precise targeting of the master regulator *JHAMT* in nymphs efficiently disrupts reproduction, which accounts for the parasitoid’s host preference.

The stage-specific regulation of the vitellogenin system further supported the adaptive advantage of parasitizing nymphs (Figure 5D). The 3rd instar nymphs only showed vitellogenin receptor (*VgR*) downregulation on day 1 (Log2FC = −0.92, though *p* = 0.199), suggesting a subtle preparatory suppression prior to the onset of vitellogenesis. This preemptive strategy in nymphs contrasts with the delayed but severe suppression observed in adults, where both vitellogenin (*Vg*; Log2FC = −0.94, *p* = 6.05 × 10^−5^) and its receptor (VgR; Log2FC = −1.68, *p* = 1.34 × 10^−2^) required coordinated downregulation by day 3 to halt active yolk protein synthesis and uptake.

Collectively, our results reveal distinct profiles for regulating the juvenile hormone and vitellogenin systems in parasitized 3rd instar nymphs versus adults, thereby providing a molecular basis for stage-specific outcomes of parasitism.

### 3.6. RT-qPCR Validation

**Figure 6:**
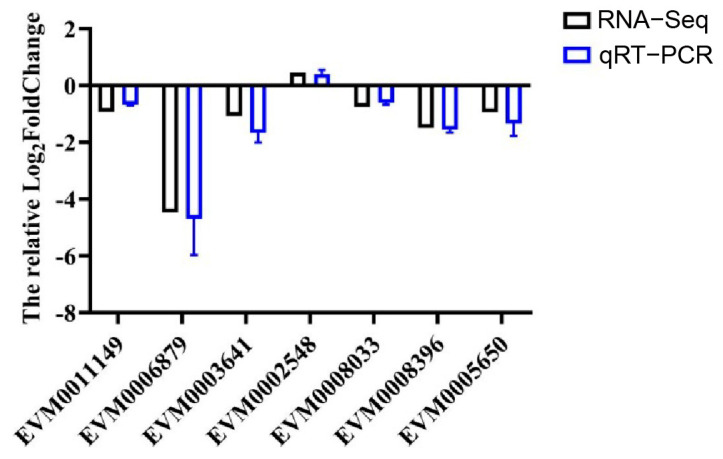
Validation of RNA-Seq data by RT-qPCR analysis.

A total of 7 differentially expressed genes (DEGs) were selected for RT-qPCR validation, including those from the insect hormone biosynthesis pathway (EVM0006879, EVM0008033, EVM0008396), the insulin signaling pathway (EVM0003641, EVM0002548), and the vitellogenin and receptor pathway (EVM0011149, EVM0005650). The relative expression levels of these genes, as determined by RT-qPCR, were consistent with the transcriptomic data from RNA-Seq (Figure 6). This consistency validated the reliability of the transcriptome sequencing data.

## 4. Discussion

The study provides comprehensive insights into the molecular mechanisms underlying *B. communis* parasitism of *A. gossypii*, revealing stage-specific strategies that optimize parasitoid fitness through differential host manipulation. Our integrated approach, which combined biological observations with transcriptomic analysis, demonstrates that *B. communis* employs distinct molecular tactics when parasitizing nymphs versus adults, with nymphs representing the optimal target for efficient reproductive suppression. Our results not only advance the understanding of parasitoid-host interactions but also identify potential molecular targets for novel pest management strategies.

The biological impacts of parasitism in our study manifested as significant developmental delays and reproductive suppression across all host stages, with severity inversely correlated with host age at parasitization. These observations have been documented in previous studies on *Lysiphlebia japonica* parasitizing *Aphis gossypii*, which prolonged development and severely suppressed reproduction [38]. Similarly, *Cotesia ruficrus* parasitization inhibited molting and pupation in *Cnaphalocrocis medinalis* [9] while *Microplitis similis* extended 3rd instar duration in *Spodoptera litura* and *S. exigua* [39]. Furthermore, the venom of *Aphidius ervi* induces ovarian apoptosis in *Acyrthosiphon pisum*, inducing apoptosis of aphid ovarian cells and causing host sterilization [40]. These findings corroborate our data showing that parasitized aphids exhibited significantly reduced offspring production. Although these results confirm parasitoids’ regulatory role in aphid development and reproduction, the underlying molecular mechanisms necessitate further exploration.

Transcriptomic analysis revealed the molecular underpinnings of these phenotypic changes, with 1168 DEGs identified across different stages and time points. The temporal progression of DEG numbers went from minimal changes after the nymphs were parasitized for 1 day (7 DEGs) to maximum changes after the adults were parasitized for 3 days (549 DEGs), which reflected the escalating molecular interplay between the parasitoid manipulation and host resistance. KEGG pathway analysis highlighted the central role of disrupted insect hormone biosynthesis. Concurrently, the activation of immune-related pathways revealed a “reproduction-immunity trade-off,” wherein hosts appear to redirect resources from reproduction to immune defense. This trade-off aligns with predictions of life-history evolution, where parasitism imposes selective pressures that compel hosts to sacrifice reproduction for survival assurance [41].

Among the differentially expressed genes, 2 genes emerged as particularly significant for understanding parasitoid reproductive manipulation strategies. *JHAMT* showed stage-specific expression patterns that illuminated why parasitoids prefer nymphs. In nymphs, targeting *JHAMT* alone (−3.23-fold change at day 3) was sufficient for efficient reproductive disruption, whereas adult parasitism required both progressive *JHAMT* suppression (fold change from −1.49 to −4.47) and the alteration of multiple other genes. Juvenile hormone (JH) can be involved in the regulation of a series of physiological processes such as growth and development, metamorphosis, reproduction, embryogenesis and caste differentiation in insects [42,43,44,45,46,47]. *JHAMT* has been reported as a key regulatory enzyme in the synthesis of *JH* in *Drosophila melanogaster* [48], *Aedes aegypti* [49], and other insects. A previous study demonstrated that *JHAMT* knockdown dramatically reduced body length, body weight, survival rate, and pupation rate in *Agrotis ipsilon* [50], which is consistent with our observations that a decrease in *JHAMT* leads to development delayed and suppressed fecundity in aphids. However, reduced *JHAMT* activity does not uniformly prolong developmental duration across insect species. In *Leptinotarsa decemlineata*, dsRNA-mediated *JHAMT* knockdown shortened developmental duration, inducing precocious metamorphosis and complete oviposition failure in females [51].

The vitellogenin (Vg) system is indispensable for egg maturation and embryogenesis, thus playing a pivotal role in insect fecundity [52,53]. In our study, the vitellogenin-vitellogenin receptor system only showed coordinated suppression in adults (both *Vg* and *VgR* downregulated by day 3), whereas nymphs exhibited subtle *VgR* downregulation before vitellogenesis onset. Since vitellogenin synthesis is primarily regulated by juvenile hormone and ecdysone [54], we propose that the suppression of the Vg system is a downstream consequence of the upstream disruption of hormone biosynthesis, particularly through *JHAMT* downregulation. This is supported by studies showing JH is a primary inducer of *Vg* in non-social insects [55] and that JH analogs upregulate Vg expression [56,57]. Therefore, the suppression of Vg/VgR in parasitized aphids likely results from upstream hormonal manipulation rather than direct targeting. This indirect regulation through hormonal manipulation represents a more efficient parasitoid strategy than targeting individual reproductive proteins, as hormones act as master regulators controlling entire gene networks. Collectively, these molecular insights demonstrated that *B. communis* has evolved to exploit a developmental window where host manipulation is most efficient—3rd instar nymphs, which possess sufficient resources for parasitoid development and lack the complex compensatory mechanisms of adults, enabling “low-cost, high-reward” parasitism through precision targeting of master regulators rather than the multi-gene assault required in adults.

In conclusion, our findings elucidate the sophisticated molecular strategies employed by *B. communis* to manipulate host reproduction. The distinct stage-specific parasitism strategies reveal a remarkable evolutionary optimization: minimal intervention in nymphs versus a comprehensive molecular assault in adults, achieving an optimal balance between parasitoid fitness and host survival. While our study focused on the *B. communis*-*A. gossypii* system, the principles uncovered here likely extend to other parasitoid-aphid interactions, suggesting convergent evolution of reproductive manipulation mechanisms. Understanding these molecular intricacies not only advances fundamental knowledge of parasitoid biology but also opens new avenues for developing biocontrol strategies that specifically target the reproductive systems of pests.

## 5. Conclusions

This study provides the first comprehensive molecular characterization of how *B. communis* parasitism affects *A. gossypii* reproduction across different developmental stages. Through integrated biological and transcriptomic analyses, we demonstrated that parasitoids employ distinct molecular strategies when exploiting nymph versus adult hosts, identifying the 3rd instar nymph stage as the optimal developmental window for efficient reproductive manipulation. We identified *JHAMT* as a master regulator that can be precisely targeted in nymphs, contrasting with the multi-gene suppression required in adults, and revealed the evolutionary refinement of parasitoid strategies that maximize reproductive control while minimizing energetic investment. These findings not only advance our understanding of the molecular basis of parasitoid-host interactions but also provide valuable insights for developing innovative pest management strategies. By revealing the natural mechanisms through which parasitoids achieve “low-cost, high-reward” host manipulation, the findings reflect how parasitoids balance energy investment and parasitic benefits during long-term evolutionary processes. They also show how natural selection shapes optimal parasitic strategies and provides crucial insights into the broader evolutionary patterns of parasitoid-host interactions.

## Figures and Tables

**Table 1:** Primer sequences for reproduction-related genes and reference gene.

Primer Name	Forward Primer Sequence (5’-3’)	Reverse Primer Sequence (5’-3’)
EVM0006879	ATGTCTGCCGTGTTTGGAAC	TGCCTCAACCGACTTGGATA
EVM0008033	CCGCAGTTCAAGACCAACAT	CCATCCGTGCAACATAAGCA
EVM0005650	TGTGGCGATAGTTCCGATGA	GTTCCGTTACACCTGGCTTT
EVM0011149	AAATCTGTTGGCTCGGTTCG	ACCTACTCAGGTCGCTGAAG
EVM0008396	AATATATAAACGTGCACAAGCG	GTTGGAGTCTGGACCTCCTT
EVM0003641	TCGGAACCGATCAAACAACG	CCGAAATGTCGGTGTTAGGG
EVM0002548	CGTGGGTGCAAATCATGGAA	TTTGCGGACATCCAACACTG
β-actin	TGGACTCTGGTGACGGTGTCTC	ATTTCTCTTTCAGCGGTGGTGG

## Data Availability

The original data presented in the study are openly available in NCBI at PRJNA1354908.
